# Extraction and Study of the Essential Oil of Copal (*Dacryodes peruviana*), an Amazonian Fruit with the Highest Yield Worldwide

**DOI:** 10.3390/plants9121658

**Published:** 2020-11-26

**Authors:** Eduardo Valarezo, Santiago Ojeda-Riascos, Luis Cartuche, Nathaly Andrade-González, Inés González-Sánchez, Miguel Angel Meneses

**Affiliations:** Departamento de Química y Ciencias Exactas, Universidad Técnica Particular de Loja, Loja 110150, Ecuador; esojeda@utpl.edu.ec (S.O.-R.); lecartuche@utpl.edu.ec (L.C.); nathyrs24@hotmail.com (N.A.-G.); iagonsalez3@gmail.com (I.G.-S.); mameneses@utpl.edu.ec (M.A.M.)

**Keywords:** *Dacryodes peruviana*, essential oil, α-phellandrene, antibacterial activity, antifungal activity, repellent activity, antioxidant activity

## Abstract

Essential oils are highly demanded substances worldwide. They can be used without modification due to their different chemical and biological properties or as natural sources of chemical compounds. The limit in the use of these metabolites is their low yield. In the present investigation, the essential oil of fruits from *Dacryodes peruviana* collected in the Ecuadorian Amazon was extracted and studied. The essential oil was released from the plant matrix and isolated by hydrodistillation. The yields obtained were 4.8 ± 0.2% and 11.3 ± 0.2% for fresh and dried fruits, respectively, one of the highest yields on record to date. Twenty-five chemical compounds were identified by GC/MS and GC/FID techniques. The principal constituent was α-phellandrene, with 50.32 ± 3.32%. The antimicrobial activity of the oil was assayed against five Gram negative bacteria, two Gram positive bacteria and two fungi. The essential oil exerted a moderate activity against *Staphylococcus aureus*. The repellent activity of the oil was assayed against mosquitoes (Diptera: Culicidae); the samples with 3%, 2% and 1% essential oil were class 4, and the sample with 0.5% showed to be class 3. The essential oil showed a weak antioxidant activity through the DPPH and ABTS methods.

## 1. Introduction

Burseraceae is a family of flowering plants belonging to order Sapindales, composed of about 18 genera, which includes over 649 currently accepted species of resinous trees and shrubs (The Plant List, 2013). Burseraceae are native primarily to tropical America with few species in Africa and Asia (Encyclopaedia Britannica, 2020). These species produce gum resins and essential oils, which are responsible of many medicinal properties (El-Shemy, 2018). From the literature, essential oils are recognized as a rich source of bioactive compounds, which present biological activities such as antifungal, antibacterial, anti-insecticidal, antiviral and antitumor; the identification of these activities has increased the interest in looking for biological properties of essential oils.

In Ecuador, a megadiverse country with approximately 17,000 species of plants, the species of the Burseraceae family represent only 0.3% of the total species, with 40 individuals, most of which are native and one is endemic [1]. Within the Burseraceae family, the *Dacryodes* genus ranks fifth in number of species with 62 individuals, after the genera *Commiphora* (208 individuals), *Bursera* (120 individuals), *Protium* (104 individuals) and *Canarium* (88 individuals) [2]. *Dacryodes peruviana* (Loes.) H.J. Lam (class: Equisetopsida C. Agardh; subclass: Magnoliidae Novák ex Takht.; superorder: Rosanae Takht.; order: Sapindales Juss. ex Bercht. & J. Presl; family: Burseraceae Kunth; genus: *Dacryodes* Vahl) is a native species of Ecuador, widely distributed in the Andean and Amazonian regions between 0 and 2500 m a.s.l., especially in the Amazonian provinces of Morona-Santiago, Napo, Pastaza, Zamora-Chinchipe [1]. The species is known as “*copal*” “*copal comestible*” and “*anime*” (Spanish language), and “*wigonkawe*” (Wao tededo, dialect of the Amazon region) [3]. The *Dacryodes peruviana* plant is a 20–25 m tall tree with alternate and odd-pinnate compound leaves, with 5–9 leaflets (12–30 × 7–11 cm). It has inflorescence in its panicle and subminal, and its flower is unisexual, with cupuliform calyx and three yellowish-green petals. The fruit is an indehiscent ovoid drupe, which is used as food for monkeys, birds and for human consumption. The resin is used as incense and mosquito repellent. The trunk is used as wood in the construction of houses, cabinetry and carpentry [3]. Currently, there are no reports of the toxicity of this species.

Currently, Ecuador occupies the sixth position worldwide in the number of plant species per unit surface area, which makes this country a biodiversity hotspot [4]. However, there are few studies of its aromatic plant species, especially of the aromatic species of the Burseraceae family; for this reason, the aims of this research are (i) to contribute to the knowledge of the aromatic species of the Burseraceae family, isolating and studying the essential oil of the species *Dacryodes peruviana* from Ecuador, (ii) to promote the use of essential oils by providing information on their availability, chemical composition and biological properties, and (iii) to provide an alternative for the use of non-timber resources, by using the fruits as a replacement for wood and leaves. In addition, as we know, this is the first report of the extraction of essential oil of this species and the study of its physical properties, chemical composition and biological activity.

## 2. Results

### 2.1. Essential Oil Extraction

**Figure 1:**
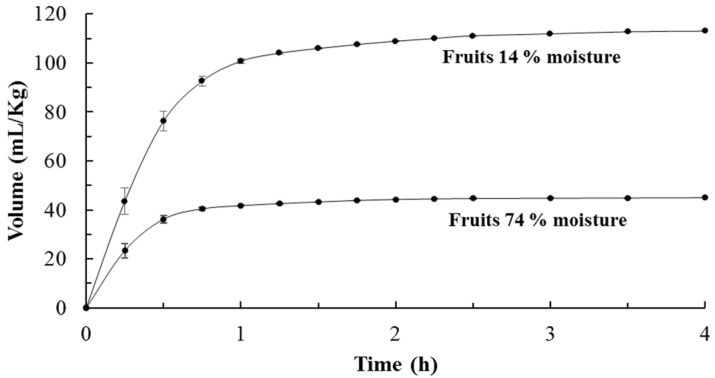
Essential oil extraction rate.

From fresh fruits, with 74 ± 3% of moisture, approximately 480 mL of essential oil (AE) from 10,000 g of vegetal material were obtained, which represented a yield of 4.8 ± 0.2% (*v*/*w*) or 48 mL/Kg. On the other hand, from 10,000 g of dried fruit, with 14 ± 1% moisture, ~1130 mL of essential oil were obtained, which represents a yield of 11.3 ± 0.3 (*v*/*w*) or 113 mL/Kg. Figure 1 shows the essential oil extraction rate (accumulated volume vs. time); the amount of essential oil extracted per hour decreases over time from 42 mL/Kg in the first hour to 0.2 mL/Kg in the last hour. In both cases (fresh and dried fruits), 90% of essential oil is obtained in 0.75 h and 95% in 1 h.

### 2.2. Physical Properties of Essential Oil

The essential oil of *D. peruviana* was a viscous liquid with a density of *d*^20^ = 0.8456 ± 0.0023 g/cm^3^, refractive index of *n*^20^ = 1.4751 ± 0.0002 and specific rotation of ${\left[\alpha \right]}_{D}^{\mathrm{20}}$ = +12.2 ± 0.7.

### 2.3. Essential Oil Compounds Identification

The identification of volatile compounds present in *Dacryodes peruviana* of Ecuador was carried out by GC/MS and GC/FID. The qualitative and quantitative data of the chemical composition of the oils of this species are shown in Table 1. In essential oil, twenty-five chemical constituents were identified, representing 99.1% of the total composition. These constituents were mainly grouped into aliphatic monoterpene hydrocarbons (ALM, 94.40%); furthermore, a low amount of oxygenated monoterpenes (OXM, 1.17%) were identified and the presence of oxygenated sesquiterpene (OXS) was not determined. The principal constituents are found to be ALM (CF: C10H16, MM: 136.13 Da) α-phellandrene (50.32 ± 3.32%), limonene (23.03 ± 2.53%), α-pinene (8.27 ± 1.28%) and terpinolene (5.23 ± 0.93%). In addition, 3.06 ± 0.80% of the ARM ρ-cymene was determined.

### 2.4. Biological Activity

In the estimation of the net activities (including synergism and antagonism) of the compounds present in the essential oil, the biological activity was determined based on their antibacterial activity, antifungal activity, and repellent and antioxidant activities.

#### 2.4.1. Antibacterial Activity

Essential oils obtained from fruit of *D. peruviana* were assessed against five Gram-negative and two Gram-positive bacteria; the MIC (minimum inhibitory concentration, μg/mL) values are shown in Table 2. The maximum concentration tested was 5000 μg/mL; the bacterium *S. typhimurium* did not provide MIC at this concentration (MIC > 5000 μg/mL), and the microorganism that presented the lowest MIC was the Gram-positive bacterium *S. aureus*.

#### 2.4.2. Antifungal Activity

The *D. peruviana* fruits essential oils were assessed against two dermatophytes fungi in order to determine its antifungal activity; the MIC (μg/mL) are shown in Table 2. The two fungi tested, *T. rubrum* and *T. mentagrophytes*, showed an activity of MIC = 2500 μg/mL.

#### 2.4.3. Repellent Activity

In order to know the repellent activity of the essential oil for possible applications as an active compound in repellent formulations, four concentrations of oil were tested; the results are shown in Table 3. In the first hour, the sample with 3% essential oil showed a PR = 100%. In all concentrations, the activity of hour 1 was higher than that of the other hours; there was no change in the PR from hour 2 to hour 5. The samples with 3%, 2% and 1% essential oil were class 4, and the sample with 0.5% was shown to be class 3. For all times, the positive control had a PR of 100% (class 5).

#### 2.4.4. Antioxidant Capacity

The ABTS radical cation and DPPH radical scavenging activity were used to explore the antioxidant activity of essential oil of *D. peruviana.* The half maximal inhibitory concentration (IC_50_) was used as a measure value of the inhibition concentration of 50% of the activity, and, as positive controls, BTH and Trolox were used.

Through the ABTS method, the essential oils showed a 27% of inhibition to 1000 ppm; a low amount of antioxidant activity. However, with the concentration ranges tested in the ABTS method, essential oil did not provide IC_50_ values (IC_50_ > 1000 μg/mL). Among the standards tested, Trolox with an IC_50_ = 460 μg/mL was the most efficient positive control. Employing the DPPH technique, a 22% of inhibition with 1000 ppm was obtained, however the essential oil did not provide IC_50_ values in the concentration ranges tested (Table 4).

## 3. Discussion

Essential oils are highly demanded substances worldwide due to their large number of chemical and biological properties [5]. The limit for the use of these metabolites is their low yield. The yield and extraction time of essential oil depends on the plant species and the part of the plant used for the extraction: leaves, trunk, fruits, roots, etc. [6]; for this reason, the yields are very varied with values < 0.01% up to values > 3%. For common genera such as Eucalyptus, yields have been reported to range from 0.2 to 2.5% for fresh plant material (leaves) [7]. Considering that essential oil is a commercially important substance, several efforts have been made to improve yields and reduce extraction times; research includes use or treatment with enzymes [8], microwave [9,10], supercritical carbon dioxide [11], steam explosion [12], etc. Extraction times by hydrodistillation range from two to four hours [9], and from one to two hours if microwave-assisted hydrodistillation is used [10]. The short extraction times in the present investigation are because the essential oil was previously released from the plant matrix. The release prior to the hydrodistillation process makes it possible to obtain the majority (95%) of the essential oil in the first hour of distillation. The authors propose that the optimal extraction time would be 1.5 h, with which it would be possible to obtain an optimal extraction amount of 98% of essential oil; 470 mL (47 mL/Kg) from fresh and 1107 mL (111 mL/Kg) from dried fruit.

According to the categorization proposed by the “Science and Technology for Development” (CYTED), for plant species the yield values in essential oil of less than 5 mL/Kg are considered low, values between 5 mL/Kg and 10 mL/Kg intermediate, and values greater than 10 mL/Kg high [13]. According to this classification, the essential oil yield of copal fruit is considered as high yield. In *Bursera graveolens* (Kunth) Triana & Planch (Burseraceae) [14], a yield of 3.7% has been reported in essential oil of sawdust from the trunks of trees, and a yield of 2.9% for fresh fruits, and in the species *Schinus molle* L. (Anacardiaceae) a yield of 3.5% of the fruits was obtained [15]. However, in the available literature, it was not possible to find a higher yield than that obtained in this study.

Most essential oils have relative densities of less than 1 (density of water) [16] and refractive indices between 1.4 and 1.6. According to Delgado Ospina et al., the refractive index in essential oils is influenced by the chemical composition of the oil, that is, the refractive index in an essential oil is an average weighting of the refractive indices of the components of the mixture [17]. Therefore, it is useful as a measure of purity, quality and chemical composition change.

The essential oil of *Dacryodes peruviana* fruits is constituted by ~50% α-phellandrene (CAS 99-83-2), which is a cyclic monoterpene that is widespread in nature and has been identified as the main compound of several essential oils [18,19,20]. α-Phellandrene has been reported to not be genotoxic in Chinese hamster ovary cells and to promote tumor formation on the skin of mice treated with the primary carcinogen DMBA (7,12-Dimethylbenz[a]anthracene), but is not carcinogenic in its own right; this compound presents minimal risk of irritation sensitization or toxicity (acute oral toxicity in rats LD_50_: 5.7 g/kg, acute dermal toxicity in rabbits LD_50_: >5g/kg) [21]. The α-phellandrene has demonstrated antioxidant [22] and insecticidal activity [19], antihyperalgesic and antidepressive actions [20], and produces antinociceptive activity in rodents [23] and promotes immune responses in mice [24]. Essential oils containing α-phellandrene as one of the main compounds have shown antioxidant, repellent [25], antimicrobial [18], antiacetylcholinesterase [26], antihyperalgesic and antidepressive activity [20].

For natural products, there are no accepted standard criteria for defining the in vitro antimicrobial activity. However, Van Vuuren and Holl [27] suggested a detailed classification scale for extracts and essential oils that are relevant to the characterization of the activity of our samples. For an essential oil, an activity ≤100 μg/mL is considered to be very strong, from 101 to 500 μg/mL the activity is strong, a moderate activity is reported when a MIC value lies between 501 to 1000 μg/mL, and activities >1001 μg/mL are considered inactive. On this basis, the essential oil of fruit from copal presented a moderate activity against Gram-positive bacterium *Staphylococcus aureus* (ATCC 25923) with a MIC of 625 μg/mL. Considering that the panel should at least consist of a Gram-positive and a Gram-negative bacterium and a fungus, the essential oil was tested against five Gram-negative and two fungi, however, the EO of copal showed activities >1000 μg/mL, 2500 (Table 2). The phenomenon of “additive” or “synergistic” effects in mixtures or extracts frequently causes loss-of-activity [28]. The antibacterial capacity against *S. aureus* of the EO from *D. peruviana* fruit may be due to its high concentration in α-phellandrene and limonene, which have been reported as compounds with antibacterial capacity [29,30].

Regarding repellent activity, all concentrations tested showed strong repellent activity [31]. The repellent activity is directly related to the chemical composition and the main compounds contained in the essential oil. Compounds similar to the main compounds found in copal EA have been reported in other essential oils with repellent activity. The essential oil of *Senecio pogonias*, in which α-phellandrene (22%), α-pinene (48%) and p-cymene (7.1%) were reported, presented a PR of 68% (Class IV) against *Triatoma infestans* Klug [25]. Essential oils with limonene and α-pinene as the main compounds, such as the essential oil of *Tagetes minuta* [32] and *Myrica gale* [33], show strong (100% and 82%, respectively) mosquito repellent activity. In addition, good repellent activity has been reported for AEs that contain high amounts of limonene such as the essential oils of *Citrus reticulata*, *Citrus limon* and *Citrus aurantium* [34]. The results obtained validate the use of *D. peruviana* essential oil as an active principle in the elaboration of repellent formulas such as cream, spray or lotions.

With the ABTS and DPPH methods, the antioxidant activity obtained was weak. For the essential oils, the weak antioxidant activity in the DPPH assay can be explained by the fact that terpene compounds are not capable of donating a hydrogen atom, and by the low solubility provided by them in the reaction medium of the assay, because this test utilizes methanol as a solvent [35]. The weak in vitro antioxidant activity of essential oils from *D. peruviana* mainly consisting of monoterpene with proven antioxidant activity, such as α-phellandrene [22] and limonene [36], may be due to antagonism with other compounds present in essential oil.

## 4. Materials and Methods

### 4.1. Materials

Dichloromethane, glycerin, methanol, sodium sulfate anhydrous, dimethyl sulfoxide (DMSO), Mueller Hinton broth (MH broth), butylated hydroxytoluene (BHT), (±)-6-Hydroxy-2,5,7,8-tetramethylchromane-2-carboxylic acid (trolox), 2,2-Diphenyl-1-picrylhydrazyl (DPPH) and 2,2′-azino-bis(3-ethylbenzothiazoline-6-sulfonic acid) diammonium salt (ABTS) were purchased from Sigma-Aldrich. Helium was purchased from INDURA Ecuador. The standard of aliphatic hydrocarbons was purchased from CHEM SERVICE under code M-TPH6X4-1ML (Diesel Range Organics Mixture #2-GRO/DRO). All chemicals were of analytical grade and used without further purifications.

### 4.2. Plant Material

**Figure 2:**
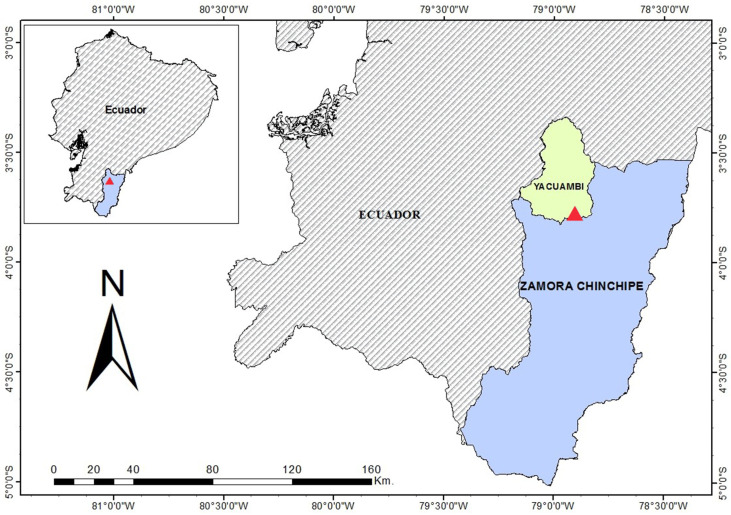
Collection sector of the fruits of *Dacryodes peruviana* in the Ecuadorian Amazon.

The fruits of the *Dacryodes peruviana* were collected in the mature state, in which most fruits have the highest volatile oil concentrations and the maximum size and weight [37], at the location La Paz parish, Yacuambi canton, Zamora Chinchipe province in the Ecuadorian Amazon (T: 25 °C and P: 0.87 atm), at a latitude of 3°40′12.8″ S and a longitude of 78°54′21.2″ W (Figure 2), and at an altitude of 1025 m a.s.l. The fruits collected were ovoid drupes 1.5 to 3 cm long with a color that was subjectively identified to be greenish yellow or reddish yellow. The plant material was collected by some of the authors under permission N° 001-IC-FLO-DBAP-VS-DRLZCH-MA granted by the Ministerio del Ambiente de Ecuador (MAE). The botanical specimens were identified by Dr. Bolivar Merino, at the herbarium of the “Universidad Nacional de Loja”.

A voucher specimen is preserved in the Herbarium of the Universidad Técnica Particular de Loja. Half of the collected fruits were dried in a drying room at 32 °C for 4–6 days, until their moisture was 14–16%. The moisture of plant material was determined using the following test method: AOAC 930.04–1930, Loss on drying (Moisture) in plants.

### 4.3. Essential Oil Extraction

For the extraction of the essential oil, two processes were carried out: release and isolation. In order to reduce extraction times and increase yields, the essential oil was first released from the vegetal matrix prior to hydrodistillation. The release of the essential oil was carried out by means of a device in the process of patenting called the “Device for the release of essential oil from a plant matrix by crushing by immersion centrifugal force” (Dispositivo para la liberación de aceite esencial de una matriz vegetal por trituración por fuerza centrífuga en inmersión), Application No. IEPI-2014-27173. The crushing of the fruit for the liberation of the essential oil was performed by centrifugal force, in immersion (water) and in vacuum, for 45 to 60 s. The release process was carried out in a single stage.

The plant material previously treated in the release device was immediately hydrodistilled in a semi-pilot distiller (80 L capacity) (Clevenger-type apparatus). The volume vs. time curve was obtained by hydrodistilling the copal fruit for four hours, collecting the essential oil for each period of time (15–30 min). Subsequently, the essential oil obtained was dried over anhydrous sodium sulphate and was stored in sealed vials, protecting them from light at 4 °C until being used in the analysis [38].

### 4.4. Determination of Physical Properties of Essential Oil

With the aims of knowing the physical nature of essential oil, some of its physical properties were determined. The density of essential oils was determined according to the standard AFNOR NF T 75–111 using a pycnometer (1 mL) and an analytical balance (model Mettler AC 100 ± 0.0001). Refraction index was determined according to the standard AFNOR NF T 75–112 using a refractometer (model ABBE). The optical rotation was determined according to the standard method ISO 592:1998 using an automatic polarimeter model Mrc-P810. The measurements were performed at 20 °C.

### 4.5. Essential Oil Compounds Identification

Qualitative analysis of essential oils was carried out by gas chromatography-mass spectrometry (GC/MS) as per the procedures described earlier by Valarezo et al. [39], using an Agilent gas chromatograph (model 6890N series) coupled to a mass spectrometer (quadrupole) detector (model Agilent series 5973 inert). Identification of the constituents was carried out by comparing the retention index (RI) and mass spectral data (MS) with those of published literature [40,41,42]. The RI were obtained through the arithmetic index described by van Den Dool and Dec Kratz [43] using the Equation (1). Quantitative analyses were performed using an Agilent gas chromatograph (model 6890N series) equipped with a flame ionization detector (GC/FID) according to the procedure described by Valarezo, Tandazo, Galán, Rosales and Benítez [39]. The relative amounts of individual components were calculated based on the GC peak area (FID response) without using a correction factor. In both cases, for GC/MS and GC/FID, a J&W DB-5ms Ultra Inert GC column (30 m, 0.25 mm, 0.25 μm) and an automatic injector (series 7683) were used. The GC column was maintained at 50 °C for the first 3 min, followed by gradient of 3 °C/min until 230 °C, which was held for 3 min (run time 66 min).

(1) $$\mathrm{RI}=100n+100\frac{\left(RTx-RTn\right)}{\left(RTN-RTn\right)}$$

where *n* is the carbon number of the hydrocarbon that elutes before the compound of interest, *RTx* is the retention time of the compound of interest, *RTn* is the retention time of the hydrocarbon that elutes before the compound of interest and *RTN* is the retention time of the hydrocarbon that elutes after of the compound of interest.

### 4.6. Biological Activity

#### 4.6.1. Evaluation of Antibacterial Activity

Antibacterial activity was evaluated against the Gram-negative bacteria *Proteus vulgaris* (ATCC 8427), *Pseudomona aeruginosa* (ATCC 27853), *Escherichia coli* (ATCC 25922), *Klebsiella pneumonia* (ATCC 9997) and *Salmonella typhimurium* (LT2), and Gram-positive bacteria *Staphylococcus aureus* (ATCC 25923) and *Enterococcus faecalis* (ATCC 29212), according to the procedure described by Valarezo, Guamán, Paguay and Meneses [38]. The bacterial strains were incubated in MH broth and the minimum inhibitory concentration (MIC) was determined by the two fold serial dilution method using 96-well microtiter plates [44]. Tetracycline was used as the positive control for Gram-negative and Gram-positive bacteria and DMSO was considered as negative control (positive growth with 5% DMSO as final concentration).

#### 4.6.2. Evaluation of Antifungal Activity

Antifungal activity was evaluated against *Trichophyton rubrum* (ATCC 28188) and *Trichophyton mentagrophytes* (ATCC 28185) by the microdilution method [45,46], as described earlier by Valarezo, Guamán, Paguay and Meneses [38]. Terbinafine was used as the positive antimycotic control and DMSO as the negative control. MIC was the lowest concentration of EO that prevented visible fungal growth.

#### 4.6.3. Evaluation of Repellent Activity

To carry out the essential oil repellency tests, the method reported by Talukder and Howse [47] and modified by Lopez, Lima, Agüero, Lopez, Hadad, Zygadlo, Caballero, Stariolo, Suero, Feresin and Tapia [25] was used, with some modifications. Filter paper (Whatman Grade 1, 90 mm diameter circles) was cut in half. In one of the filter paper halves, 1 mL of different concentrations of a solution of essential oil and glycerin was applied homogeneously; the concentrations of the solution were 0.5%, 1%, 2% and 3% of essential oil (*v*/*w*). One mL of glycerin was placed in the other half of the filter paper. The treated filter papers were allowed to air dry until the solvent evaporated, then the two filter paper semicircles were placed contiguously in a Petri dish. Inside the Petri dish, 10 mosquitoes (Diptera: Culicidae) were released. The count of mosquitoes present in each half was carried out every hour for five hours and a commercial repellent with active ingredient N, N-Diethyl-meta-toluamide (DEET) was used as a positive control. To convert the results into a percentage of repulsion (PR), the following formula is applied: PR (%) = (Nc − 50) × 2, where Nc is the percentage of mosquitoes present in the half where the glycerin was applied (control half). Positive values (+) indicate repellency and negative values (−) indicate attraction. All tests were repeated three times and mean values were classified according to Table 5 [47].

#### 4.6.4. Evaluation of Antioxidant capacity

##### DPPH Radical Scavenging Activity

The DPPH free radical scavenging activity of oils was measured based on the scavenging activity of the stabilized 2,2-diphenyl-1-picrylhydryl radical, the method has been described previously by Valarezo et al. [48]. A solution of DPPH was prepared and the absorbance adjusted with methanol until to obtain a reading of 1.1 ± 0.02 absorbance units at a wavelength of 515 nm in an UV spectrophotometer (Genesys 10S UV-Vis Spectrophotometer, Thermo Scientific). Quantities of 150 μL of the essential oil diluted at different concentrations (0.025 to 2.5 mg/mL) were mixed with 2850 μL of DPPH and allowed to react for 24 h at room temperature, protected from light; after this, the absorbance was measured at 515 nm.

The same amount of methanol was added instead of the sample solution as a blank control, and BHT and Trolox were used as positive controls. The percentage of free radical-scavenging capacity was calculated using the following formula:

(2) $$\mathrm{Radical}\;\mathrm{scavenging}\;\left(\%\right)=\left[\frac{A_{s}-A_{i}}{A_{s}}\right]\times 100$$

where *Ai* is the absorbance of the samples and *As* is the absorbance of blank control. The essential oil concentration providing 50% inhibition (IC_50_) was calculated from the graph by plotting inhibition% against sample concentration.

##### ABTS Radical Cation Scavenging Activity

The ABTS assay [49] with some modifications [50] was used to evaluate the free radical scavenging of essential oil as described by Valarezo et al. (2020). The ABTS^•+^ radical cation standard solution was prepared by reaction of ABTS and potassium persulfate; this solution was diluted in methanol until an absorbance of 1.1 ± 0.02 was obtained in a spectrophotometer UV with a wavelength of 734 nm. Quantities of 150 μL of the essential oil diluted at different concentrations (0.025 to 2.5 mg/mL) were mixed with 2850 μL of ABTS and allowed to react for 2 h at room temperature, protected from light, and immediately the absorbance was measured at 734 nm. The same amount of deionized water was used as blank control while BHT and Trolox were used as positive controls. The inhibition of ABTS^•+^ radicals was determined as follows:

(3) $$\mathrm{Radical}\;\mathrm{scavenging}\;\left(\%\right)=\left[\frac{A_{o}-A_{i}}{A_{o}}\right]\times 100$$

where *Ao* is the blank control absorbance and *Ai* is the absorbance of the samples. The essential oil concentration providing 50% inhibition (IC_50_) was calculated from the graph by plotting inhibition% against sample concentration.

### 4.7. Statistical Analysis

The analyses of moisture, physical properties, repellent activity and antioxidant capacity were performed in triplicate. The procedures of essential oil extraction, compound identification and antibacterial and antifungal activity were repeated nine times. Data were collected in Microsoft Excel; measures of central tendency and standard deviation were calculated using Minitab 17 (Minitab^®^ 17.3.1. Statistical software, State College, PA, USA). All results are expressed as mean values.

## 5. Conclusions

This is the first report on the extraction, chemical composition and biological activity of essential oil from this species. The results of the present study demonstrated the high yield of essential oil in these species, making the copal fruit a suitable option for industrial exploitation. α-Phellandrene, limonene, α-pinene and terpinolene were the main components. The in vitro biological activities showed that essential oil exerted a moderate activity against *Staphylococcus aureus*. The essential oil of *Dacryodes peruviana* showed strong repellent activity against mosquitoes. The results obtained in the present investigation give a strong argument for the use of copal fruits, a resource that is renewed annually, as an alternative to the use of trees for wood.

## 6. Patents

“Device for the release of essential oil from a plant matrix by crushing by immersion centrifugal force" (Dispositivo para la liberación de aceite esencial de una matriz vegetal por trituración por fuerza centrífuga en inmersión), Application No. IEPI-2014-27173, INSTITUTO ECUATORIANO DE PROPIEDAD INTELECTUAL (IEPI).

## Figures and Tables

**Table 1:** Chemical composition of essential oil from *Dacryodes peruviana.*

Peak #	Compound ^a^	RI	RI^ref^	*D. peruviana*		Type	CF	MM
				*%* ^b^	*SD*			(Da)
1	Tricyclene	921	921	0.05	0.03	ALM	C_10_H_16_	136.13
2	α-Thujene	926	924	1.90	0.14	ALM	C_10_H_16_	136.13
3	α-Pinene	932	932	8.27	1.28	ALM	C_10_H_16_	136.13
4	Camphene	947	946	0.13	0.04	ALM	C_10_H_16_	136.13
5	Sabinene	969	969	1.44	0.40	ALM	C_10_H_16_	136.13
6	β-Pinene	973	974	2.57	0.91	ALM	C_10_H_16_	136.13
7	Myrcene	986	988	0.73	0.20	ALM	C_10_H_16_	136.13
8	α-Phellandrene	1005	1002	50.32	3.32	ALM	C_10_H_16_	136.13
9	δ-3-Carene	1010	1008	0.18	0.11	ALM	C_10_H_16_	136.13
10	α-Terpinene	1013	1014	0.32	0.05	ALM	C_10_H_16_	136.13
11	ρ-Cymene	1021	1020	3.06	0.80	ARM	C_10_H_14_	134.10
12	Limonene	1025	1024	23.03	2.53	ALM	C_10_H_16_	136.13
13	γ-Terpinene	1055	1054	0.23	0.24	ALM	C_10_H_16_	136.13
14	Terpinolene	1082	1086	5.23	0.93	ALM	C_10_H_16_	136.13
15	Camphor	1140	1141	tr	-	OXM	C_10_H_16_O	152.12
16	Terpinen-4-ol	1170	1174	0.07	0.05	OXM	C_10_H_18_O	154.14
17	γ-Terpineol	1202	1199	0.98	0.31	OXM	C_10_H_18_O	154.14
18	Ascaridole	1236	1234	0.12	0.02	OXM	C_10_H_16_O_2_	168.12
19	δ-Elemene	1338	1335	0.06	0.07	ALS	C_15_H_24_	204.19
20	α-Copaene	1366	1374	0.05	0.03	ALS	C_15_H_24_	204.19
21	trans-Caryophyllene	1415	1417	0.13	0.09	ALS	C_15_H_24_	204.19
22	α-Humulene	1451	1452	tr	-	ALS	C_15_H_24_	204.19
23	Germacrene D	1476	1480	0.15	0.01	ALS	C_15_H_24_	204.19
24	δ-Amorphene	1509	1511	tr	-	ALS	C_15_H_24_	204.19
25	β-Curcumene	1514	1514	0.07	0.09	ALS	C_15_H_24_	204.19
*Aliphatic monoterpene hydrocarbons (ALM)*				94.40				
*Aromatic monoterpene hydrocarbons (ARM)*				3.06				
*Oxygenated monoterpenes (OXM)*				1.17				
*Aliphatic sesquiterpene hydrocarbons (ALS)*				0.47				
Total identified				99.10				

^a^ Compounds ordered according to the elution order in the column DB5-Ms; RI. retention indices in the a-polar column (DB5-Ms); RI^ref^. references: Adams 2007, NIST 05 2005 and NIST 2020; ^b^ Percentage values are means of nine determinations; tr. trace (<0.05%); -. not detected; CF. Chemical Formula; MM. Monoisotopic mass.

**Table 2:** Antibacterial and antifungal activity of essential oil of copal (*Dacryodes peruviana*)*,* given as minimal inhibitory concentration (MIC, μg/mL).

Microorganism	*D. Peruviana*	Positive Control ^b^
	MIC (μg/mL) ^a^	
**Gram-Negative Bacteria**		
*Pseudomonas aeruginosa* (ATCC 27853)	5000	3.91
*Klebsiella pneumoniae* (ATCC 9997)	2500	1.95
*Proteus vulgaris* (ATCC 8427)	2500	3.91
*Escherichia coli* (ATCC 25922)	2500	1.95
*Salmonella typhimurium* (LT2)	>5000	1.95
**Gram-positive Bacteria**		
*Enterococcus faecalis* (ATCC 29212)	2500	15.62
*Staphylococcus aureus* (ATCC 25923)	625	15.62
**Dermatophytes Fungi**		
*Trichophyton rubrum* (ATCC 28188)	2500	20
*Trichophyton mentagrophytes* (ATCC 28185)	2500	20

^a^ Mean of nine determinations; ^b^ Tetracycline for all bacteria and Terbinafine for fungi.

**Table 3:** Repellent activity of essential oil from *Dacryodes peruviana.*

Essential Oil Concentration	Repellency (%) ^a^					Mean Repellency	Class Repellency
	1 h	2 h	3 h	4 h	5 h ^b^		
3%	100	60	60	60	60	70%	4
2%	80	60	60	60	60	65%	4
1%	80	60	60	60	60	65%	4
0.5%	60	40	40	40	40	45%	3
Control (+) ^c^	100	100	100	100	100	100%	5

^a^ Percentage of repellency PR(%) = (Nc − 50) * 2, where Nc is the percentage of mosquitoes present in the control half; ^b^ hours after treatment; ^c^ commercial repellent, active ingredient N, N-Diethyl-meta-toluamide (DEET).

**Table 4:** Antioxidant activity of essential oils of *Dacryodes peruviana.*

Sample	DPPH	ABTS
	IC_50_^a^ (μg/mL)	
*D. peruviana EO*	>1000	> 1000
BHT	430 ± 30	290 ± 20
Trolox	460 ± 50	260 ± 30

^a^ IC_50_ = Inhibition Concentration of 50%.

**Table 5:** Classification scale (Class) of repellent activity according to repellency rate (PR,%)

Class	Repellency Rate (%)
0	>0.01 to <0.1
1	0.1 to 20
2	20.1 to 40
3	40.1 to 60
4	60.1 to 80
5	80.1 to 100
